# The power of people who use drugs as mass media influencers in changing public opinion during the global overdose epidemic

**DOI:** 10.1186/s13011-026-00709-6

**Published:** 2026-02-13

**Authors:** Ehsan Jozaghi, Vancouver Area Network of Drug Users (Vancouver Area Network of Drug Users is a consortium/not-for-profit)

**Affiliations:** 1 The University of British Columbia, Vancouver, BC V6T 1Z3 Canada; 2 Vancouver Area Network of Drug Users, 380 E Hastings St, Vancouver, BC V6A 1P4 Canada

**Keywords:** Mass media, VANDU, Harm reduction, Social media, People who use drugs, Addiction, Advocacy, Activism

## Abstract

**Background:**

Thousands of people have lost their lives due to overdose-related fatalities linked to the potent synthetic opioid epidemic, as mortalities increase annually. British Columbia (B.C.) Canada has been an exception, where the population not only has more liberal views on illegal drugs, but overdose death numbers have reduced for the first time in recent months. The reduction of overdose deaths in BC and liberal views have taken place in conjunction with the Vancouver Area Network of Drug Users’ (VANDU) advocacy growth over the past two decades.

**Methods:**

The Canadian Newsstream database, which contains news media coverage, was utilized to examine VANDU’s news coverage and impact in 2023. VANDU’s mass media impact is determined qualitatively. NVivo 14 facilitated coding 97 articles in 2023, out of 1,596 total news articles since 1997.

**Results:**

After analyzing 97 articles from 42 blogs, 33 newspapers, 21 wire feeds, and one other source, five themes were identified. These themes included (1) policy engagement, (2) Government lobbying, (3) Advocacy, (4) Unsanctioned work, and (5) Demonstration.

**Conclusion:**

With the rise of pseudoscience and anti-harm reduction rhetoric across the globe, this research has shown that partnering and funding drug user advocacy groups is the most critical way to fight the overdose epidemic because the advocacy can change public opinion and therefore government policy that needlessly contributes to preventable deaths.

## Introduction

Vancouver Area Network of Drug Users (VANDU) was founded in the 1990s in the Downtown Eastside (DTES) of Vancouver, British Columbia (BC) by a group of former, current, and community advocates to counter the growing overdose deaths, blood-borne infections, and other related illnesses affecting people who use drugs (PWUDS) in the face of government inaction at both municipal, provincial, and federal levels [1, 2]. While initially operating with a small grant from the local health board, VANDU now has a modest operating budget to support a storefront and various advocacy and harm-reduction programs that extend beyond needle exchange and overdose prevention sites [3]. For instance, VANDU now supports many low-barrier programs, including, but not limited to, the Western Aboriginal Harm Reduction Society (WAHRS), Tuesday’s education group, the British Columbia Association for People on Opioid Maintenance (BCAPOM), and the Eastside Illicit Drinkers Group for Education (EIDGE) [3]. VANDU has also launched a new group, “Our Streets,” in response to city sweeps that displace people living in tents in the DTES [4]. Some of VANDU’s funding for its harm reduction groups comes from research grants, fundraising efforts, and donations [4].

Since its inception, VANDU membership has pioneered groundbreaking harm-reduction projects, including unsanctioned needle depots, unsanctioned injection sites, unsanctioned inhalation rooms, injection support teams, safe supply/compassionate clubs, and demonstrations [1, 2, 5–11]. Moreover, research has discussed VANDU’s various collaborations and pioneering research activities over the past two decades [12]. However, no study has addressed the impact of VANDU’s media engagement on public opinion through a qualitative inquiry [13]. This is particularly true, as recent work has highlighted the important role of framing in shaping public opinion on controversial topics, such as harm reduction [14]. In effect, Canadian newspapers’ media attention of harm reduction and drug users based on previous research “independently shaped public discourse, frequently characterizing harm reduction” via various perspectives (e.g., criminal justice or health), biased or framed [14, 15].

An integral part of mass media is ‘framing’ opinion and thoughts about a topic, which are so well established that researchers view its role as “a factor so deeply involved and of such central concern that its elimination would radically and fundamentally alter the very character” of society [15, p. 285]. In addition to ‘framing,’ mass media also have enormous power to set ‘agenda setting’ and alter laws or polices by choosing to reflect on a particular issue for reporting [15]. Thus, it is not surprising that many harm-reduction supervised consumption sites in Ontario and other jurisdictions have been planned for a shutdown; there is a growing propaganda against harm reduction; at the same time, a movement known as forced treatment has gained momentum to replace harm reduction [16–20].

Therefore, by relying on VANDU’s mass media engagement via the Canadian Newstream database, this research presents findings on VANDU’s membership activism and advocacy through the mass media and its potential impact. It is ultimately argued that influential people with extensive social capital and long-standing media engagement are not the only ones capable of shaping or framing topics and thus changing public opinion; rather, people in the DTES can, through media engagement, influence the broader social structure and frame media topics affecting their health [21].

## Literature review on advocacy and media engagement

Social justice movements increasingly use traditional media and social media to advocate for and influence public policy, shaping public opinion to promote social and legal change [22]. By highlighting at-risk group voices, strategically highlighting issues, and energizing movement through both traditional and digital media platforms, the social justice movements can amplify their voices and mobilize their bases [22]. This type of media mobilization and activism not only raises awareness of various social justice plights but also increases political pressure on politicians, cities, and government policymakers, thereby creating opportunities for incremental change aligned with the movement’s goals [22]. The effectiveness of such media public relations campaigns and advocacy is directly linked to the relentless ability to engage diverse audiences, simple messaging, and credibility [22].

Social justice movements and their media activism are centred on the centrality of at-risk groups not only in policy-making decisions but also in advocacy through media engagement and civil disobedience [23, 24]. Previous research, for instance, has found that PWUD have been well-positioned to meaningfully advocate and lobby for their issues through groundbreaking research, whether through conceptualization or study design, and later publicize the research through media press releases [11]. In effect, “nothing about us, without us” is a statement normalized by the Canadian Legal Network of HIV/AIDS in 2005 that many at-risk and vulnerable groups, including PWUDs, have utilized to emphasize their critical role in any advocacy, public engagement, civil disobedience, and lobbying [25].

The Canadian HIV/AIDS Legal Network has been an advocacy group that has fought for the human dignity and rights of people affected by or living with HIV here in Canada and around the world for decades through the following strategies: 1-analysis and research, 2-educating the public, 3-mobilizing the community, and 4- legal advocacy and litigation in courts [26]. For instance, the Canadian HIV/AIDS Legal Network has been involved in numerous legal challenges to laws that discriminate based on HIV status which contributes to the stigma around HIV diagnosis, including the Supreme Court of Canada judgement in 2022 in R. v. Kirkpatrick which challenged the HIV non-disclosure and the 2024 legal challenge in the Federal court to the *Immigration and Refugee Protection Act*, which can deny permits based on HIV status [27, 28].

Similar advocacy groups that have a tremendous impact on mass media, policy, the legal framework, and government legislation include the Gay Liberation Front (GLF). The GLF became prominent in mass media after the 1969 Stonewall uprising, where it relied on a combination of civil disobedience, lobbying, and advocacy to challenge legal and ultimately societal discrimination and stigma around homosexuality based on human rights [29–34]. The GLF activism and civil disobedience not only shifted public opinion from tolerance toward acceptance but also eventually helped to change laws around sodomy, the criminalization of public same-sex intimacy, and the legality of marriage of same-sex couples in many countries around the world [29–34].

Similar advocacy and civil disobedience can be observed in the Sex work arena, where peer-based organizations such as Providing Alternatives, Counselling & Education (PACE) Society in Vancouver have been involved in numerous groundbreaking court cases as interveners to revamp laws governing sex work in Canada [35]. PACE Society and many similar advocacy groups use their online presence to target audiences to educate the public and change public opinion regarding the legitimacy of sex work as a profession [36]. Moreover, previous research has found that sex work advocacy groups’ websites, combined with social media platforms’ presence by peer-sex workers as social media influencers, “augment the untapped potential for creating action, mobilization, interaction, and dialogue” [36, p. 2]. Similarly, Drug User Liberation Front (DULF) was founded by two Vancouver activists in 2020 on the premise of providing its members with safe, tested drugs (e.g., illegal drugs purchased are tested via NMR and chromatography) through compassionate clubs [10]. Since the legislative changes in BC that decriminalized personal use of illegal drugs for certain amounts, DULF has been at the forefront of activism and lobbying, attempting to apply for exceptions for their compassionate club without success [37–40]. The co-founders have teamed up with external researchers to evaluate the compassionate club program, which has shown significant success in reducing fatalities linked to unregulated drug supply, reducing reliance on the illegal market, improving the well-being of membership, while promoting membership autonomy through access to a stable drug supply, and creating a sense of community membership [37–40]. Like many other activist movements, DULF co-founders have been convicted recently of drug trafficking and have thus initiated a constitutional challenge about laws governing the compassionate drug clubs that could potentially be decided by Canada’s highest court [41].

In line with many groups mentioned above, VANDU has been linked to activism and civil disobedience associated with Canada’s drug laws that historically criminalized addiction as a relapsing medical condition [42]. In effect, previous research has found that VANDU has changed the fabric of life in the DTES for many at-risk and vulnerable citizens through their relentless activism, political activism, and media engagement [1, 43, 44]. For instance, Jozaghi [43] suggested that the peer-based model of VANDU generated significant momentum, enabling many vulnerable citizens to become active in the political and public domains through acts of civil disobedience, forming a united front to confront potential discrimination by the police and in hospitals.

## Research questions and rationale

While numerous studies have examined peer-based programs and their effects on the health and well-being of at-risk groups, as discussed in the literature review, no peer-reviewed study has examined the qualitative outcomes of VANDU in the mass media, as described in the 2022 study protocol published in the *Health and Justice* journal [21]. Moreover, a quantitative extension of the noted study protocol, Jozaghi and VANDU’s [13] study, showed that as the drug overdose cases and rates spiked, so did advocacy and media engagement by VANDU, where the overdose rates and cases were a strong predictor of media engagement and activism by VANDU membership via correlation and regression analysis. Thus, it is essential to examine and complement Jozaghi & VANDU’s [13] results with rich descriptions of details and events as reflected in the mass media. Such powerful descriptions could include some of the themes, impact, content, and quotes that VANDU has been able to shape through examining the following questions:

1. What are the main themes of VANDU media impact and engagement in 2023, the year that minor drug possession was legalized for personal consumption in BC?
2. Do the identified themes convey the level of advocacy and media engagement that could influence and potentially change public opinions?

## Methods

### Data source

Two thousand members of VANDU, in the DTES of Vancouver, Canada, annually elect a board of directors [1]. What is unique about VANDU is its partnerships with local health agencies, whose core budget is funded directly by the Vancouver Health Authority [5, 6]. The organization now operates a storefront and runs various groundbreaking research, education, and outreach programs aimed directly at people in the DTES and beyond [3, 12, 13]. However, a significant part of VANDU’s work has been its advocacy via mass and social media engagement. While VANDU owns media accounts (e.g., Instagram, X, and Facebook) and podcasts (e.g., Crackdown), mass media engagement is the most important and arguably the least researched area [45].

Therefore, this research examines publicly available news sources via the ProQuest Canadian Newsstream for the impact of activism, media engagement, and community action [21]. The Newsstream was selected as a database, rather than other news sources (e.g., Google News, Factiva, LexisNexis, JSTOR, or Periodicals Archive), because it is Canadian and its archival data goes to the 1970s, which includes many newspapers, newswires, websites, videos, and magazines [214]. The ProQuest Canadian database currently houses many national and international news sources (e.g., more than 300 unique outlets) [21].

### Data collection

The main goal of data collection from ProQuest Newsstream is to identify news reports that VANDU members have shaped through their engagement, lobbying, and advocacy [21]. As seen in Table 1, more than 1,596 news items have been shaped by VANDU membership from 1997 to 2024 (e.g., 1,064 newspapers, 267 Blogs, Podcasts, and websites, 247 wire feeds, one trade journal, and 17 other article sources). The inclusion criteria included all 2023 articles that interviewed a VANDU member or reported on instances in which members’ actions and activism made headlines (e.g., demonstrations or protests).

**Table 1:** The frequency of news items obtained from the proquest Canadian newstream about VANDU

Time Ranges Start	Time Range End	Mass Media Results
1997-01-01	1997-12-31	4
1998-01-01	1998-12-31	11
1999-01-01	1999-12-31	15
2000-01-01	2000-12-31	49
2001-01-01	2001-12-31	32
2002-01-01	2002-12-31	100
2003-01-01	2003-12-31	106
2004-01-01	2004-12-31	66
2005-01-01	2005-12-31	69
2006-01-01	2006-12-31	55
2007-01-01	2007-12-31	33
2008-01-01	2008-12-31	84
2009-01-01	2009-12-31	43
2010-01-01	2010-12-31	56
2011-01-01	2011-12-31	50
2012-01-01	2012-12-31	42
2013-01-01	2013-12-31	45
2014-01-01	2014-12-31	36
2015-01-01	2015-12-31	23
2016-01-01	2016-12-31	68
2017-01-01	2017-12-31	55
2018-01-01	2018-12-31	44
2019-01-01	2019-12-31	47
2020-01-01	2020-12-31	63
2021-01-01	2021-12-31	96
2022-01-01	2022-12-31	130
2023-01-01	2023-12-31	97
2024-01-01	2024-12-31	77
**Total**		**1596**
**µ**		**57**
**σ**		**29.67**

The year 2023 was selected because it marked a significant milestone: the BC government decriminalized personal drug use [46]. It is also important to emphasize that community-based research is time-consuming; since the publication of the study protocol linked to this work in 2022, the leading author has been a full-time dentistry student with limited funding or time to support the peer researchers [21]. The word “Vancouver area network of drug users” formed the search query, which involved downloading news sources from 2023 into NVivo 14. The selected items were reviewed to assure they met the benchmarks and that duplicate articles attributed to the Newswire were not included again in the content analysis [13, 21].

## Material and data analysis

Unlike quantitative research, qualitative research can focus on interactions and unique, unusual, and unexpected cases [47]. This research relied on NVivo 14 rather than Atlas. ti and MaxQDA because of its ease of use [47–50]. Using NVivo 14, laborious coding and content analysis were simplified rather than relying on rudimentary colour coding with highlighters [47]. Therefore, after uploading the articles, inductive or open coding was used for thematic analysis, and the qualitative data were systematically analyzed. Thus, the open or inductive coding method involved identifying themes and establishing nodes. The new nodes representing themes, in line with Fereday and Muir-Cochrane [51], “involve recognizing (seeing) an important moment and encoding it (seeing it as something) … [before] a process of interpretation” (p. 83). Twelve nodes were initially created, as shown in Table 2.

**Table 2:** The inductive coding of themes, explanation, and sources

Theme	Explanation	Example
Harm reduction	Efforts in promoting harm reduction programs for at-risk people	*The Vancouver Sun* article on Feb 03, 2023, titled “B.C.‘s harm-reduction and addiction…”
Discrimination	Efforts that highlight discrimination toward PWUDs and people of the DTES	*The Canadian Press* article on Feb 01, 2023, titled “Drug users say B.C. ‘fight continues…”
Human rights advocacy	Media engagement that highlights human rights issues of PWUDs and people of the DTES	The Vancouver Sun article on Sep 15, 2023, titled, “Inside the battle to open Insite …”
DTES advocacy	Efforts that promoted the plight of the DTES people	*The Vancouver Sun* article on Apr 11, 2023, titled, “Residents from deadly Vancouver…”
Housing advocacy	Media engagements that promoted housing and the challenges of street sweeps	*The Canadian Press* article on Apr 06, 2023, titled “BR-Tent-Encampment-Vcr”
Policy engagement	Media engagements that highlighted changes in health, city, or criminal justice policy	*The Globe and Mail* article on Sep 14, 2023, titled, “Insite, North American’s first…”
Decriminalization and safe supply	Efforts that promoted decriminalization and compassionate clubs	*The Globe and Mail* article on May 26, 2023, titled “Pierre Poilievre is at war with…”
Legal advocacy	Efforts that highlighted challenges to existing laws or the current legal framework through the courts	*The Vancouver Sun* article on Feb 01, 2023, titled “Drug users say the ‘fight continues…”
Unsanctioned work	Efforts that highlighted unsanctioned efforts	*The Vancouver Sun* article on Oct 23, 2023, titled " Two arrested in connection”
Demonstration	Coverage and engagement with media during demonstrations	*The Canadian Press* article on Nov 10th, 2023, titled, “DULF, as we knew it, is dead”
Government engagement	Media coverage that highlighted VANDU’s government lobbying	*The Vancouver Sun* article on Nov 25th, 2023, titled “This Vancouver compassion…”
French article	Articles written in French	*La Presse Canadienne* article on 29 May, 2023 titled, “Une motion conservatrice défaite…”

The lead author was involved in the coding process because no funding was available to support a dignified peer-based research project. After the codes were developed through inductive methods, a deductive approach was initiated, based on six previously developed categories derived from dialogue with some VANDU board members and the first author’s own codebook, developed by watching news and social media posts throughout 2023 related to drug policy in BC and Canada, as seen in Table 3.

**Table 3:** The deductive coding themes, explanation and examples

Theme	Explanation	Example
Decriminalization	VANDU’s media linked to BC government’s decriminalization of personal drug use	*The Tyee* article on Sep 21, 2023, titled, “Where municipalities are at …”
Homelessness	VANDU’s media activities linked to street sweeps	*The National Post* article on Apr 05, 2023, titled, “Tents and suitcases go into trash…”
Demonstration	VANDU’s various demonstrations	*The Canadian Press* article on Nov 10th, 2023, titled, “DULF, as We Knew It, Is Dead”
Funding	Funding cuts to some of VANDU’s programs	*The Hamilton Spectator* article on Feb 21, 2023, titled, “Policing and politics are not a good mix”
Forced treatment	VANDU’s opposition to the expansion of forced treatment programs	*Toronto Star* article on 25 Apr 2023, titled, “Health experts urge action on drug crisis: Approach…”
Unsanctioned work	VANDU’s support for DULF and safe supply	*The Globe & Mail* article on Oct 26, 2023, titled, “Vancouver police raid drug activists’ …”

**Fig. 1:**
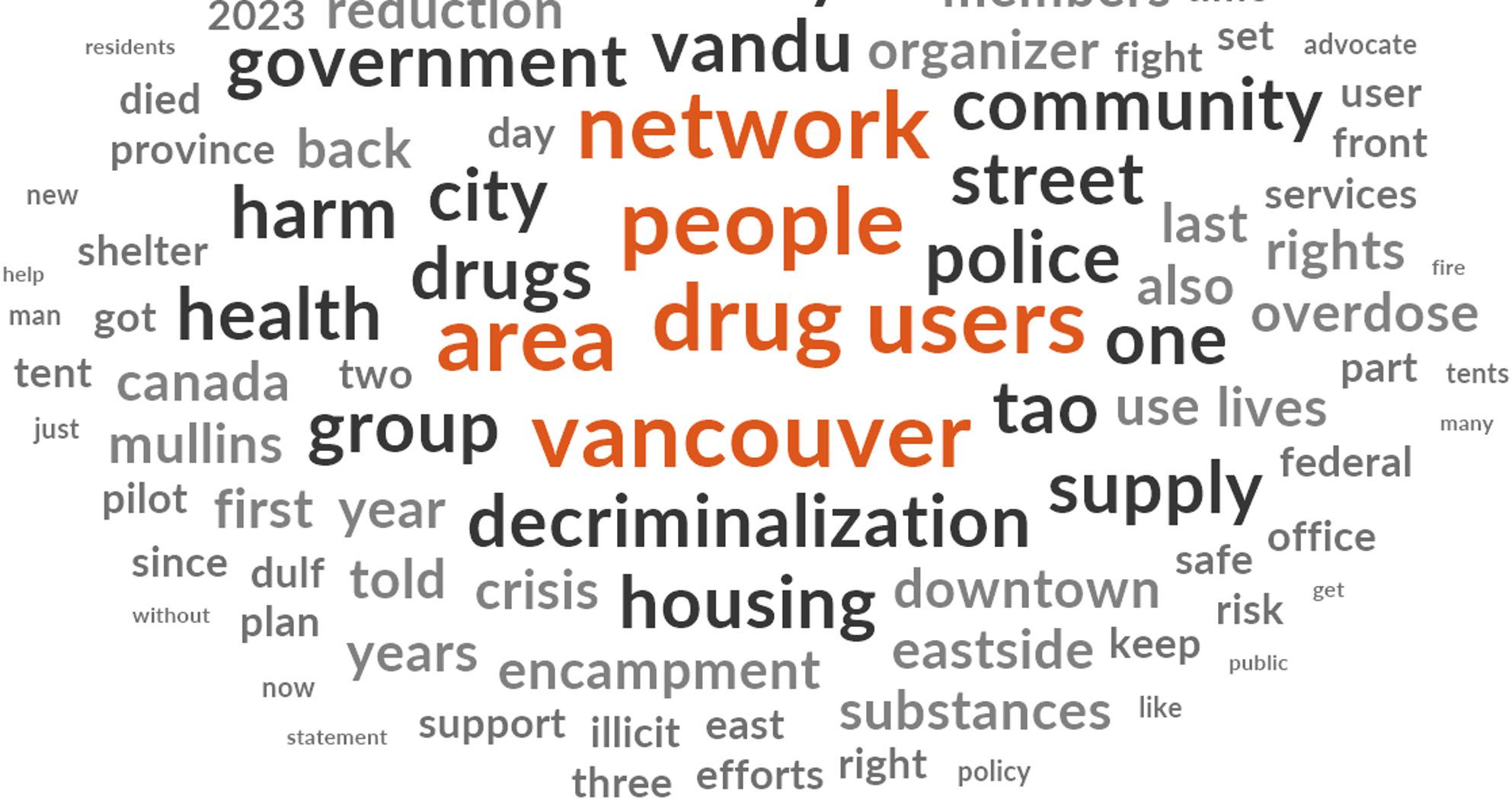
The results from the word frequency query

Thus, after the deductive coding, in which themes were identified through textual analysis based on previously identified key issues, a thematic analysis was conducted iteratively, in which the overall theme was disaggregated into sections, rearranged into different nodes, and regrouped again to create new nodes. The Word Frequency Query in NVivo was also used for further deductive coding process based on the six previously identified themes, as seen in Fig. 1.

The most frequent words and phrases were used as templates for further development in conjunction with the first author over a decade of fieldwork with the organization. Coding was conducted using a pre-constructed codebook [52]. The Node Explorer was used to create nodes before the researcher coded phrases or words in an inferential process [47]. Therefore, the deductive and inductive processes of coding, rearrangement, and amalgamation, after consultation with the VANDU board, produced five major themes, as seen in the findings section.

Validity and reliability, although essential, are not the only concepts; instead, reflexivity, credibility, transferability, confirmability, and dependability are also essential. To account for reflexivity, reflexive journaling and memos were used throughout the research process, in which the research process, actions, and assumptions were considered [53]. These assumptions included continuously examining my beliefs, background, emotions, assumptions, and interactions on the topic in a personal journal, which influenced the research project as an external researcher who has worked with VANDU for over a decade. In other words, a reflexive content analysis technique ensured that the researcher’s biases did not affect the content analysis or research decisions [54].

To ensure dependability and reliability, rather than using inter-coder reliability, this research relied on intra-code reliability (code recoding), re-examining the data and analyzing it again later to ensure consistency, dependability and reliability [55]. In qualitative interview forms, the results can also be confirmed through a “validation interview” [56]; this study’s content analysis was conducted by consulting with VANDU board members, some of whom have served on the board for over a decade with lots of mass media engagement to ensure triangulation and credibility [53]. Finally, to ensure transferability in this research, based on Lincoln & Guba [53], the thick descriptions, including quotations and descriptions of both inductive and deductive nodes or themes, are presented in Tables 2 and 3.

## Findings

### Policy engagement

VANDU’s activism and civil disobedience are oriented toward informing the public about scientific evidence and the reality of government policies shaping their lives. This is particularly true with the recent anti-harm reduction rhetoric and the forced treatment movement across Canada [16, 17]. For instance, the media statement quoted from a VANDU member below clearly demonstrates this goal:

We desperately need more evidence-based treatment programs . but if we focus on treatment and abstinence-based approaches only, we are going to leave lots of people behind. […] Harm-reduction measures are a way to support people who use drugs so they can stay healthy and alive. I’ve personally seen the [harm reduction] huge difference it makes in people’s lives.

Furthermore, the Ontario government’s promise to shut down supervised injection facilities has motivated many members to highlight the scientific evidence and effectiveness behind such peer-based projects with the media, as stated below:

Also, how the Vancouver Area Network of Drug Users’ Manifesto (vandureplace. wordpress.com/research/vandu-manifesto) outlines policies and practices on how to improve the lives of people who use drugs through user-based peer support and education to help people who use drugs live healthy, productive lives.

Many members have gone so far as to highlight their own lived experiences to showcase the ineffectiveness of the new push for forced treatment initiatives by conservative governments at the federal and provincial levels [19, 57, 58]. For example, a member of VANDU highlights his experience with such programs below:

I went to 12-step (programs). I went to all these things. That sh- didn’t work for me. I never got abstinent and clean, […] You end up with that problem if you’ve been abstinent for a while, then you slip, and your tolerance has changed, and you die.

Many VANDU members also highlighted low-barrier access to safe supply and low-barrier housing to reduce the overdose cases linked to social determinants of health, as stated below:

If we just think that sending people to treatment is going to be the answer, then we are going to fail more people again. […] But equally important is second-stage housing to support people after treatment and publicly funded counseling to deal with the underlying trauma related to the addiction.

In effect, it is not surprising that housing instability, hunger, evictions, and street sweeps increase drug-related harms and overdose risk among drug users [59–62].

### Government lobbying and engagement

VANDU’s inception in 1997 was in response to the government’s inaction on blood-borne infections and overdose deaths [1, 43]. VANDU’s lobbying and efforts at the provincial, city, and federal branches contributed to the initiation of many ground-breaking harm reduction programs in the past two decades, as summarized by a community organizer with VANDU:

A community organizer with VANDU told about 30 people packed into a room that … [VANDU has been] advocating for decriminalization for its entire 25-year history. It was also involved in a legal battles to keep open Insite.

Their lobbying effort could be observed during the recent advocacy for higher threshold limits proposed for decriminalization efforts by British Columbia (BC) and the Canadian federal government, where the VANDU organization teamed up with other groups throughout the country, as seen in the news coverage below:

“We will, with the support of our partners, friends, and allies, keep track of this experiment [of decriminalization] in our lives.” Members of VANDU, who were at the “core planning table” of meetings on decriminalization for about a year with others including Moms Stop the Harm, police and the B.C. government, suggested 18 g as a threshold [for decriminalization].

VANDU’s strategy for lobbying has always been through partnerships with other like-minded organizations via active engagement with media via press releases, as seen in the summary of the recent media blitz about the leader of the federal Conservative Party’s opposition to the safe supply program:

A coalition of groups that advocate on behalf of drug users in B.C. released a statement, voicing concern about hydromorphone, one of the drug alternatives Poilievre has singled out as problematic. The joint statement from organizations, including the Vancouver Area Network of Drug Users, says such prescriptions “help many of us reduce or eliminate our reliance on street drugs.” “If we get cut off, our risks will go up.”

Moreover, VANDU is actively involved in lobbying the municipal government for funding and programs affecting their membership, as seen in the news coverage of lobbying the Vancouver City Council:

“They punished VANDU, so I’m not surprised,” […] referring to the ABC councillors’ decision in January to reject staff’s recommendation to award a $7,500 grant to support an art program run by the Vancouver Area Network of Drug Users, or VANDU.

Unsurprisingly, Vancouver City has continued to engage and consult with VANDU on various harm reduction initiatives and projects over the past decades because, as one member stated in the news coverage, “The Downtown Eastside knows what they want and knows what they need to save lives.” The Brooms project has been the City and VANDU initiative, where “instead of city workers doing “Sweeps,” […] the people who live on these streets have been able to make $20 for an hour of cleanup”. 

### *Advocacy on behalf of the vulnerable*

In addition to policy engagement and government lobbying on drug policy issues, VANDU has advocated for anti-poverty and homelessness initiatives over the past few decades. This advocacy can be observed in the news coverage of VANDU members advocating against the ‘street sweep’ by the city of Vancouver, as seen in the news coverage below:

Many people displaced by the street sweep have nowhere else to go. “We’re trying to replace people’s tents and replace their belongings, but we’ve lost track of a bunch of people, and we’re very concerned and worried for them.” […] he was “appalled” as he watched crews who “destroyed the homes of a bunch of unsheltered people.” “There’s not enough shelter space, there’s definitely no permanent housing for people and yet they have to come down with an iron fist on the Downtown Eastside.”

Therefore, it is unsurprising that VANDU regularly issues press releases based on news coverage, as observed after the recent housing announcements by the Provincial Minister of Housing and Mayor of Vancouver, where VANDU demanded: “an end to […] street sweeps to remove people from a Downtown Eastside encampment”.

Moreover, VANDU is involved in other housing advocacy work in the neighbourhood. For example, VANDU helped in announcing a press release for a civil suit for those who lost their housing due to fire, as seen in the news coverage below:

A former resident of the Winters Hotel in Gastown is spearheading a legal action against the owner and manager of the hotel that burned to the ground last year with no fire prevention systems in place. Two people died.

But VANDU’s activism goes beyond simple news releases or media engagement; VANDU’s goal is to affect housing and criminal justice policy that disproportionately affect its membership, specifically the DTES people who use drugs, as seen in the quotation below from a news article:

Why don’t they have the money to set up an area where we can have an actual homeless camp? Set up the infrastructure, set up portable toilets, set up electricity, all that kind of stuff,” […] An organizer with the Vancouver Area Network of Drug Users said the organization found only two shelter beds available tonight, leaving people living in tents with nowhere else to go. They will come right back onto the block.

### Unsanctioned activities

When policy engagement, lobbying, and advocacy are ineffective, and the potential for death and spread of diseases is imminent, VANDU members have been involved in health, social, and civil disobedience, as evidenced in the news coverage below:

The idea of a sanctioned supervised consumption site percolated. Mr. Wilson became president of the VANDU and advocated for a site alongside community organizer Ann Livingston, poet and activist Bud Osborn, PHS executives, […] and Dan Small. They marched in the streets, attended health and police board meetings and flew to Ottawa to lobby policymakers. Critics balked at the idea, seeing it as state-sanctioned drug use. Meanwhile, Ms. Livingston paid out of pocket to open several small-scale and short-lived clandestine sites in the neighbourhood, both to support drug users and provoke authorities. “Without civil disobedience, you get absolutely nothing,” she said.

VANDU’s civil, health, and social disobedience has continued throughout the decades. An injection support team, an unsanctioned smoking facility, an unsanctioned injection facility, and more than three dozen groundbreaking research activities are evidence of this growing health civil disobedience that constantly pushes the boundaries of legal and political frameworks [3, 5, 6, 12].

The Drug User Liberation Front (DULF) [member] told the gathering she would continue her tradition of handing out free, tested heroin, cocaine, and methamphetamine as she does every time the overdose numbers are updated.

Tuesday marked the 13th “Dope on Arrival” giveaway, [… she] said as people walked up to a table one by one to claim a package of drugs when their name was called.

“We are taking these drugs as a way to prove the community can control its own safer supply,” she said. “Here’s the real tragedy. If we don’t regulate the drug supply, people will die. And I’m telling you, do not use alone, especially if you’re an opioid user.” The group has continued selling drugs, bought on the dark web, through a compassion club [where drugs are tested…] despite a rejection last year of its exemption [by Health Canada].

In such unsanctioned activities as public health civil disobedience, VANDU’s members continue to risk arrest and prison sentences, yet have collectively pushed the boundaries of the legal and criminal justice framework [2]. For such actions, VANDU has received awards in the past [12], but its cofounder has also recently received an honorary doctorate, as demonstrated in the news article below:

Ann Livingston, who will receive an honorary Doctor of Laws degree on June 16, is best known for her role in co-founding the [VANDU and] as its executive program director for 10 years. She was also a volunteer project co-ordinator for the Nanaimo Area Network of Drug Users, and helped form associations in Surrey, Abbotsford and across Canada, including the B.C. Association of People on Opiate Maintenance, Western Aboriginal Harm Reduction Society and the Canadian Association of People Who Use Drugs.

The 24-hour unlimited and unsanctioned syringe distribution, the first unsanctioned supervised injection facility, the first unsanctioned smoking facility, and various harm reduction initiatives have placed VANDU for the global struggle for more human rights and human dignity “in situations of apparent impossibility” both politically, but also legally for people with substance use disorders [2]. It is not surprising that Ann Livingston describes such unsanctioned activities as “tough fight[s]. [With recognition] that every single [harm reduction] site in every single neighbourhood of every single city will have to be fought for individually.”

### *Demonstration*s

Aside from unsanctioned activities to change public opinions and challenge the political and legal framework, VANDU and its membership have been highly effective in raising stakes for local, provincial, and federal politicians via demonstrations, rallies, and public gatherings for maximum press effects, as seen in the below newspaper coverage:

The rally, which drew around 400 participants, marched with a giant red banner which read “13,000 dead. Safe supply now.” Love on the Downtown Eastside is “powerful, because it is earned,” […] “We should not be afraid to express our hatred […for] profit from our suffering.” Critiques of for-profit, abstinence-only treatment centres were repeated throughout the rally, as was the sentiment that “unjust laws should be broken.” In [… their] speech, […they] read the names of 18 [VANDU] members who have died in the last year due to toxic drugs. Some people were openly sobbing.

In effect, some Canadian provinces have shown interest in corporate abstinence and forced treatment rather than scientifically proven harm reduction programs [18]. VANDU’s demonstrations are events that draw people from all walks of life, including politicians, lawyers, and community activists, as seen in the news coverage below:

Members of VANDU attend a rally at Oppenheimer to mark International Overdose Awareness Day in Vancouver. This was an emotional scene following a march memorializing drug overdose victims. […] “When laws are unjust, it is essential to break them,” a lawyer and executive director of the Canadian Drug Policy Coalition, told the gathered crowd. [the lawyer] cited a recent report by the UN High Commissioner for Human Rights, which called for the decriminalization and regulation of drugs that centre health and human rights.

At the highest level there is recognition that it is drug laws, not drugs, causing harm.

As part of the plan to reduce the overwhelming number of overdose deaths, the BC Government got approval from the federal government to begin experimenting with decriminalization, and VANDU once again was at the forefront via gatherings and meetings seen below:

A community organizer with VANDU told [membership that] the pilot is “just a foot in the door” for the group, which has been advocating for decriminalization.

The fight continues,” told the gathering. Report back, right to this room,” he said of the group’s efforts to compile a database of people’s experiences. “We will, with the support of our partners, friends, and allies, keep track of this experiment in our lives for the next three years.

The recent drop in mortalities in BC, when compared to other jurisdictions in Canada and internationally, becomes significant when there is a continuous and alarming rise annually in opioid mortalities. The BC concurrent successful experiment with decriminalization, harm reduction, and safe supply will potentially become more evident in the coming year as the safe supply expands in BC.

## Discussion

This is the first study to illustrate the impact and power of mass media engagement by the longest-operating North American drug user advocacy. VANDU and its members have been relentless in their efforts to change public opinions through mass media engagement. Mass media coverage of VANDU membership, with 1,596 articles in newspapers, magazines, and other outlets, could have contributed to policy changes that are currently reflected in the province being the only one in Canada with an active decriminalization scheme and safe supply programs [63]. Previous research has also suggested that “media coverage shapes and reflects views on contentious harm reduction services” [14, p.1]. Thus, partnerships among cities, public health, and vulnerable populations are effective strategies for building trust, improving social determinants of health, and cultivating positive relationships that can save lives [43, 64, 65].

The decriminalization experiment in BC, combined with the safe supply program where health care professionals (e.g., physicians, nurses [RN, and registered psychiatric nurses]) prescribe drug substitutes to the toxic and unregulated illegal drug market, has had remarkable results; in fact, drug overdose deaths in the province has reduced by 9% in very short period [66, 67]. The above statement is backed with evidence from recent research, the most recent being a peer-reviewed published research in the *Canadian Medical Association Journal*, which highlighted that “providing a safe supply of substances, including opioids such as hydromorphone and fentanyl, and psychostimulants, may reduce the risk of overdose death, decrease other harms of substance use and support patient engagement in care” [68]. Therefore, it can be argued that VANDU funding through the health agency and fundraising has created social and mass media influencers who continuously engage with mass media and social media (e.g., podcasts, Facebook, and X) to shape public opinions around drug policy based on science and evidence from their lived experiences, rather than pseudoscience propaganda. Therefore, it is not surprising that many VANDU members adamantly oppose the forced treatment agenda being advocated by many conservative politicians, both federally and provincially, not only based on their lived experience shown in the findings section of this study, but through numerous peer-reviewed studies in abstinence and forced treatment that show low retention rates in such treatment model programs [69].

A 2023 systematic review in the *Canadian Journal of Addiction* examined the effectiveness of involuntary treatment, drawing on a thorough review of more than 40 peer-reviewed studies [70]. The study found that only a single study reported success in post-treatment substance use reduction, which, over the long term, was unsustainable [70]. VANDU membership, on the other hand, advocates for permanent housing through mass media, based on their lived experience and numerous studies in the field [71]. Previous peer-reviewed work has indicated that unhoused people have a greater risk of overdose when compared to adequately housed people [72]. Moreover, areas with higher eviction rates had significantly higher overdose death rates than areas with fewer evictions [73]. Previous research has also pointed out that street sweeps “contribute to less effective management of chronic health conditions, infectious diseases, and substance use disorders, and may increase physical injuries and worsen mental health.” [74]. Unsurprisingly, many researchers have argued that housing is a fundamental human rights issue that tremendously influences health (mental and physical) and other health determinants [75, 76].

In effect, the rise of radical influencers of the past century based on pseudoscience and reliance on new form of media, such as Hitler (social Darwinism), Lenin (bolshevism), Putin (land expansionism), and Khomeini (Shi’a religious hegemony)—has shown the impact of long-term mass and now social media engagement in framing topics, political or international issues and thus public opinions [77].

VANDU membership, as a mass media influencer, has demonstrated that even at-risk and underprivileged individuals have the power to engage media, lobby the government for policy change, advocate for the vulnerable, push the boundaries of the legal framework through unsanctioned activities, and ultimately change the world. The current study’s findings are like a Canadian study examining harm reduction coverage in the mass media, which described such coverage as “rarely negative in tone and consistently focused on health perspectives on harm reduction” rather than criminal justice [14, p. 8]. Therefore, it can be argued that VANDU, as mass media influencers, has increasingly framed drug policy not as a criminal justice issue that can be solved by legal enforcement or forced treatment but rather as a personal health issue that can only be remedied via safe supply, decriminalization, social housing, and harm reduction.

### Strengths and limitations

This is the first in-depth qualitative evaluation of the mass media impact of a drug user advocacy group worldwide. The long-term relationship between the first author, as an external researcher, and VANDU enhanced the validity and reliability of the content analysis. Moreover, VANDU’s board’s corroboration of the findings is another strength of these results, reinforcing their validity and reliability. However, this study reports only on a short time frame (e.g., 2023 or 97 articles). Incorporating other years could have generated additional themes linked to issues VANDU has advocated over the past decades, such as access to clean needles, rising rates of blood-borne infections, court cases, and other more interesting themes or avenues. Furthermore, this research does not conduct sentiment analysis to examine the potential negative perception of VANDU in other news databases (e.g., Google News, Factiva, LexisNexis, JSTOR, or Periodicals Archive). Therefore, this research does not aim to achieve representation; instead, its primary goal was to provide a glimpse of VANDU’s engagement in mass media over a single year. In fact, “there is a place for a small … [qualitative] study to make [a] meaningful contribution to knowledge … [since] rare situations are often precisely what the researcher wants.” [78, 79]. In other words, the primary objectives of qualitative studies are not generalizations, but rather understanding the processes, mechanisms, and nuances that make the sample unique [78, 79]. Finally, it is essential to note that VANDU is the only drug user advocacy organization examined in Canada, and there are a dozen such organizations across Canada, either established or in the process of being established. Thus, this research has neglected to account for the combined effects of other drug users’ advocacy groups that promote harm reduction and human rights in social and mass media.

## Conclusion

Since its inception in 1997 by a community activist and a poet, VANDU and its membership have pushed and reframed legal, social, and public opinion in BC and across Canada on issues affecting its membership. Their activism, advocacy, policy engagement, unsanctioned work, and demonstrations have redefined the legal and policy framework that previously brought significant sorrow and loss to its membership. The VANDU, the longest-running drug advocacy group in the world, has contributed thousands of articles to the mass media through engagement. VANDU’s mass media engagement has focused on policy, lobbying, advocacy, demonstrations, and challenging laws through unsanctioned work and public health civil disobedience, potentially saving thousands of lives by shaping public opinion and, in turn, government policy. The VANDU case study demonstrates that advocacy and media engagement can potentially change public opinion and, in the long term, contribute to changes in government laws and policies.

VANDU’s case study on mass media engagement has proven that societal change is achieved only through hard work, sacrifice, and activism, not by mysterious forces.

## Data Availability

All data generated or analyzed during this study are included in this published article (e.g., tables and figures).

## Abbreviations

BC: British Columbia
BCAPOM: British Columbia Association for People on Opioid Maintenance
DTES: Downtown Eastside
EIDGE: Eastside Illicit Drinkers Group for Education
VANDU: Vancouver Area Network of Drug Users
WHARS: Western Aboriginal Harm Reduction Society
